# Association between gestational age and child health and neurodevelopment in twins from a nationwide longitudinal survey in Japan

**DOI:** 10.1038/s41598-025-24186-2

**Published:** 2025-11-18

**Authors:** Kei Tamai, Naomi Matsumoto, Akihito Takeuchi, Makoto Nakamura, Misao Kageyama, Takashi Yorifuji

**Affiliations:** 1 Division of Neonatology, NHO Okayama Medical Center, 1711-1 Tamasu, Kita-ku, Okayama, 701-1192 Japan; 2 Department of Epidemiology, Faculty of Medicine, Dentistry and Pharmaceutical Sciences, Okayama University, Okayama, Japan

**Keywords:** Behavioral development, Child health, Early term, Gestational age, Hospitalization, Multiple births, Medical research, Epidemiology, Paediatric research

## Abstract

Despite previous research, evidence on the relationship between gestational age and long-term health and neurodevelopmental outcomes among twins remains limited. Using data from the Longitudinal Survey of Babies in the 21st Century, we analyzed 549 twins born in Japan in 2010. The twins were grouped by gestational age: <32 weeks (very preterm), 32–36 weeks (moderately and late preterm), and 37–38 weeks (early term). The health status was evaluated by hospitalization between 0.5 and 5.5 years, and behavioral development was assessed using questionnaires at 2.5 and 5.5 years. Binomial log-linear regression with generalized estimating equations accounted for within-pair correlations and adjusted for child and parental variables. Moderately and late preterm children showed a higher risk of all-cause hospitalization during infancy than early-term children (adjusted risk ratio, 1.7; 95% CI, 1.0–2.6). Very preterm children showed a higher point estimate of the risk ratio, but a wide CI (risk ratio, 2.3; 95% CI, 0.8–6.8). Behavioral delays were more common in preterm groups at 2.5 years but not at 5.5 years. Preterm twins have a higher risk of hospitalization during infancy and developmental delay at 2.5 years than early-term twins. These risks show an increasing trend as gestational age decreases.

**Supplementary Information:**

The online version contains supplementary material available at 10.1038/s41598-025-24186-2.

## Introduction

Twin pregnancies are associated with a higher rate of preterm birth than singleton pregnancies^1^. Several studies have examined the associations between gestational age in twins and short-term outcomes for both infants and mothers^2–4^. A systematic review suggested that delivery at 37 weeks in dichorionic twins and 36 weeks in monochorionic twins optimally balances the risk of neonatal mortality and stillbirth^5^. On the basis of these findings, many countries, including Japan, have developed guidelines for the timing of twin delivery, tailored to their specific national contexts^6–8^. However, the long-term neurodevelopmental impact of gestational age in twins remains insufficiently explored, particularly in contemporary cohorts. We previously reported that twins born at early term (37–38 gestational weeks) in 2001 had the lowest risks of childhood hospitalization and adverse behavioral development at 2.5 years of age^9^. Similarly, a population-based cohort study in Scotland (*n* = 43,133 twins) showed that twins born before 37 weeks of gestation between 1980 and 2015 had a higher risk of requiring special education in elementary school than those born at 37 weeks^10^. Nonetheless, most studies focused on children born around 2000, often covering a broad range of birth years, and data on more recent cohorts remain limited.

The first edition of the Guidelines for Obstetrics Practice in Japan, introduced in 2008, emphasized increased attention to twins at or after 37 weeks for fetal well-being^11^. However, the guideline did not specify a preferred gestational age for twin delivery, and its recommendations primarily focus on perinatal management rather than long-term child outcomes. Meanwhile, the preterm birth rate for twins has gradually increased over the past few decades, rising from 29.4% in 1980 to 50.3% in 2019^1^. Similar trends have been observed in the United States, Europe, and Australia, largely driven by increases in iatrogenic preterm birth^12–14^. In addition, the use of antenatal corticosteroids for preterm infants became well-established between 2000 and 2010^15^ and was increasingly adopted for both singleton and twin pregnancies^16,17^. Given these shifts in clinical practice and birth trends, examining more contemporary cohorts is crucial for gaining an updated understanding of the relationship between gestational age and long-term neurodevelopmental outcomes in twins.

To address this gap in knowledge, this study examined the association between gestational age and child health and neurodevelopment among twins born in 2010, using data from a longitudinal, population-based survey that assessed behavioral development at ages 2.5 and 5.5 years.

## Methods

### Study design, setting, and participants

The Ministry of Health, Labour, and Welfare launched the Longitudinal Survey of Babies in the 21st Century to investigate Japan’s declining birth rate^18–21^. This survey included 43,767 children born between May 10 and May 24, 2010, representing approximately 1/24 of all births in Japan that year. The initial questionnaire was administered when the children were 6 months of age, and subsequent annual questionnaires were sent to the guardians of 38,554 children (response rate at 6 months of age: 88.1%) until the children reached 5.5 years of age. The sixth survey was completed in 2015. Birth record data, such as birth weight, gestational age, singleton or multiple births, sex, parity, and parental age at delivery, from the Vital Statistics system, were also obtained for each child included in this survey.

**Fig. 1:**
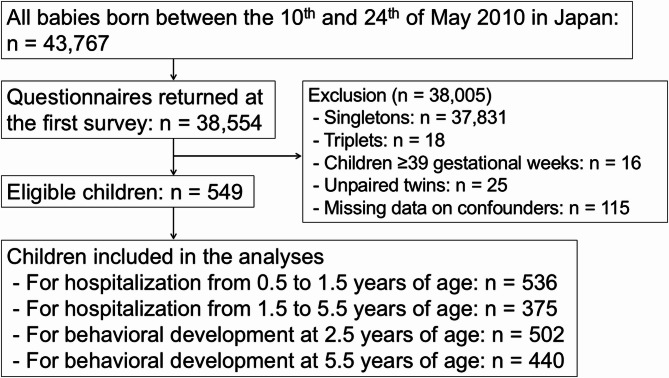
Flowchart of the participants.

Singletons (*n* = 37,831) and triplets (*n* = 18) were excluded (Fig. 1). Children born at or after 39 weeks of gestation (*n* = 16) were also excluded because twin births at this gestational age have become increasingly rare in Japan owing to the trend of earlier delivery timing for twins^22^. Unpaired twins (*n* = 25) were excluded because analyses required data from both twins to account for within-pair similarities and accurately assess twin-related outcomes. Children with missing data on confounders (*n* = 115) were excluded to perform complete-case analysis.

This study was conducted in line with the principles of the Declaration of Helsinki. Approval was granted by the Institutional Review Board of the Okayama University Graduate School of Medicine, Dentistry and Pharmaceutical Sciences (No. 1506-073). Due to the retrospective nature of the study, the Institutional Review Board of the Okayama University Graduate School of Medicine, Dentistry and Pharmaceutical Sciences waived the requirement to obtain informed consent. All data were anonymized and collected in accordance with applicable ethical guidelines and regulations.

### Gestational age

We obtained each child’s gestational age from the birth record. Gestational age ranged from 25 weeks to 38 weeks. We categorized the participants into three groups according to gestational age: <32 weeks (very preterm), 32–36 weeks (moderately and late preterm), and 37–38 weeks (early term). These groups were based on a previous study that assessed the associations between gestational age and child health and neurodevelopment in singleton pregnancies, as well as recommendations regarding the definition of “term” pregnancies from the American College of Obstetricians and Gynecologists^23^.

### Child health and behavioral development outcomes

To examine the effect of gestational age on long-term morbidity among twins, we used overnight hospitalizations in early childhood (up to 5.5 years of age) as an indicator of health status. The survey obtained information on whether the child had been hospitalized during the previous 12 months for any reason and asked respondents to provide further details if the hospitalization resulted from one of several common diseases. The same question was asked in each survey from 1.5 to 5.5 years of age. In this study, we focused on hospitalizations that occurred during infancy (i.e., 0.5–1.5 years) and early childhood (i.e., 1.5–5.5 years). We analyzed hospitalizations from all causes and specifically focused on those caused by respiratory infections, as they are the most common reason for hospitalization in childhood.

The survey queried behavioral development at 2.5 and 5.5 years of age. We assessed the effect of gestational age on the neurodevelopment status among twins using behavioral development at both of these ages. In the survey, the parents were asked to provide a “yes” or “no” response according to whether children had reached various age-appropriate motor, language, and behavioral milestones. The questions used to assess behavioral development have not been externally validated or taken from an established scale, which should be considered when interpreting the findings. Nevertheless, they have been used in previous studies to assess risk factors for unfavorable development^20,21,24^.

At 2.5 years of age, the following questions were asked: (1) Can your child walk?, (2) Can your child run?, (3) Can your child climb stairs?, (4) Can your child say things that make sense?, (5) Can your child compose two-phrase sentences?, (6) Can your child say his or her name?, and (7) Can your child use a spoon to eat? At 5.5 years of age, the following questions were asked: (1) Can your child listen carefully?, (2) Can your child focus on one task?, (3) Does your child remain patient?, (4) Can your child express emotions appropriately?, (5) Can your child get along with others in a group setting?, and (6) Can your child keep promises?

### Statistical analysis

We first conducted a descriptive analysis of children categorized by gestational age: <32 weeks (very preterm), 32–36 weeks (moderately and late preterm), and 37–38 weeks (early term). We also evaluated the baseline characteristics of children with and without missing data on confounders, and of those with and without follow-up data at age 2.5 years. We then performed a binomial log-linear regression analysis to evaluate the relationships between the gestational age categories defined above and overnight hospitalizations and behavioral developments. We accounted for correlations within the pairs with the generalized estimating equation. The original dataset does not specify the twin pairs. Therefore, we identified the pairs on the basis of the birthplace and parents’ birthdays from the birth record. We estimated the risk ratio (RR) and 95% confidence interval (CI) for the main outcomes using the early-term category (i.e., 37–38 weeks) as a reference and adjusted for child and parental factors.

We selected the following potential confounders according to previous studies: sex (binary), equal sex pair (binary), small for gestational age (binary), parity (0 or ≥ 1, binary), maternal age at delivery (< 25 years, 25–34 years, and ≥ 35 years; categorical), maternal smoking during pregnancy (non-smoker and smoker, binary), maternal educational level, and place of birth and residence (ward, city, and town/village; categorical)^9,10,25^. We classified the educational level into three categories as follows: high school or less, junior college (2 years) or vocational school, and university (4 years) or higher. Small for gestational age was defined as a birth weight below the 10th percentile for gestational age, according to standard Japanese neonatal anthropometric charts^26^. If model convergence was not achieved, covariates considered to have weak associations with the outcomes or those suspected of multicollinearity were excluded from the analysis.

In the sensitivity analyses, we further categorized the moderately and late preterm group into two subcategories (32–33 weeks and 34–36 weeks) and repeated the analysis using the same reference group (37–38 weeks) to examine the effect of moderately and late preterm birth in more detail.

Stata SE version 18 statistical software (StataCorp., College Station, TX, USA) was used for all analyses.

## Results

Table 1 shows the baseline characteristics of 549 eligible children with categorization by gestational age. The very preterm group (< 32 weeks) included fewer children than the other groups. Children born very preterm and moderately to late preterm were more likely to have same-sex twins, young mothers, and mothers who smoked than those born early term.

**Table 1:** Demographic characteristics of children included in the analysis.

	Gestational age category			
	< 32 weeks	32–36 weeks	37–38 weeks	All
	(*N* = 22)	(*N* = 271)	(*N* = 256)	(*N* = 549)
Sex				
Male	9 (40.9)	134 (49.4)	127 (49.6)	270 (49.2)
Female	13 (59.1)	137 (50.6)	129 (50.4)	279 (50.8)
Sex concordance of twin pairs				
Same sex	16 (72.7)	207 (76.4)	178 (69.5)	401 (73.0)
Different sex	6 (27.3)	64 (23.6)	78 (30.5)	148 (27.0)
Parity				
Primipara	18 (81.8)	141 (52.0)	134 (52.3)	293 (53.4)
Multipara	4 (18.2)	130 (48.0)	122 (47.7)	256 (46.6)
Small for gestational age	9 (40.9)	84 (31.0)	72 (28.1)	165 (30.1)
Maternal age categories				
<25 years	2 (9.1)	20 (7.4)	4 (1.6)	26 (4.7)
25–34 years	6 (27.3)	158 (58.3)	156 (60.9)	320 (58.3)
≥35 years	14 (63.6)	93 (34.3)	96 (37.5)	203 (37.0)
Maternal smoking during pregnancy	2 (9.1)	16 (5.9)	8 (3.1)	26 (4.7)
Maternal educational attainment				
University graduate or higher	6 (27.3)	66 (24.4)	74 (28.9)	146 (26.6)
Vocational school/junior college graduate	10 (45.5)	131 (48.3)	120 (46.9)	261 (47.5)
High school graduate or below	6 (27.3)	74 (27.3)	62 (24.2)	142 (25.9)
Residential area				
Wards	6 (27.3)	80 (29.5)	92 (35.9)	178 (32.4)
Cities	14 (63.6)	161 (59.4)	142 (55.5)	317 (57.7)
Towns and villages	2 (9.1)	30 (11.1)	22 (8.6)	54 (9.8)

Categorical variables are shown as numbers (%).

Supplementary Table 1 presents the characteristics of children with and without missing data on confounders, and Supplementary Table 2 shows the characteristics of children with and without follow-up data at 2.5 years of age. Missing data were primarily due to incomplete information on maternal educational attainment, and such children were more likely to have younger mothers and mothers who smoked. Children without follow-up data were more likely to have younger mothers, mothers who smoked, and mothers with lower education levels than those with follow-up data at 2.5 years of age.

**Fig. 2:**
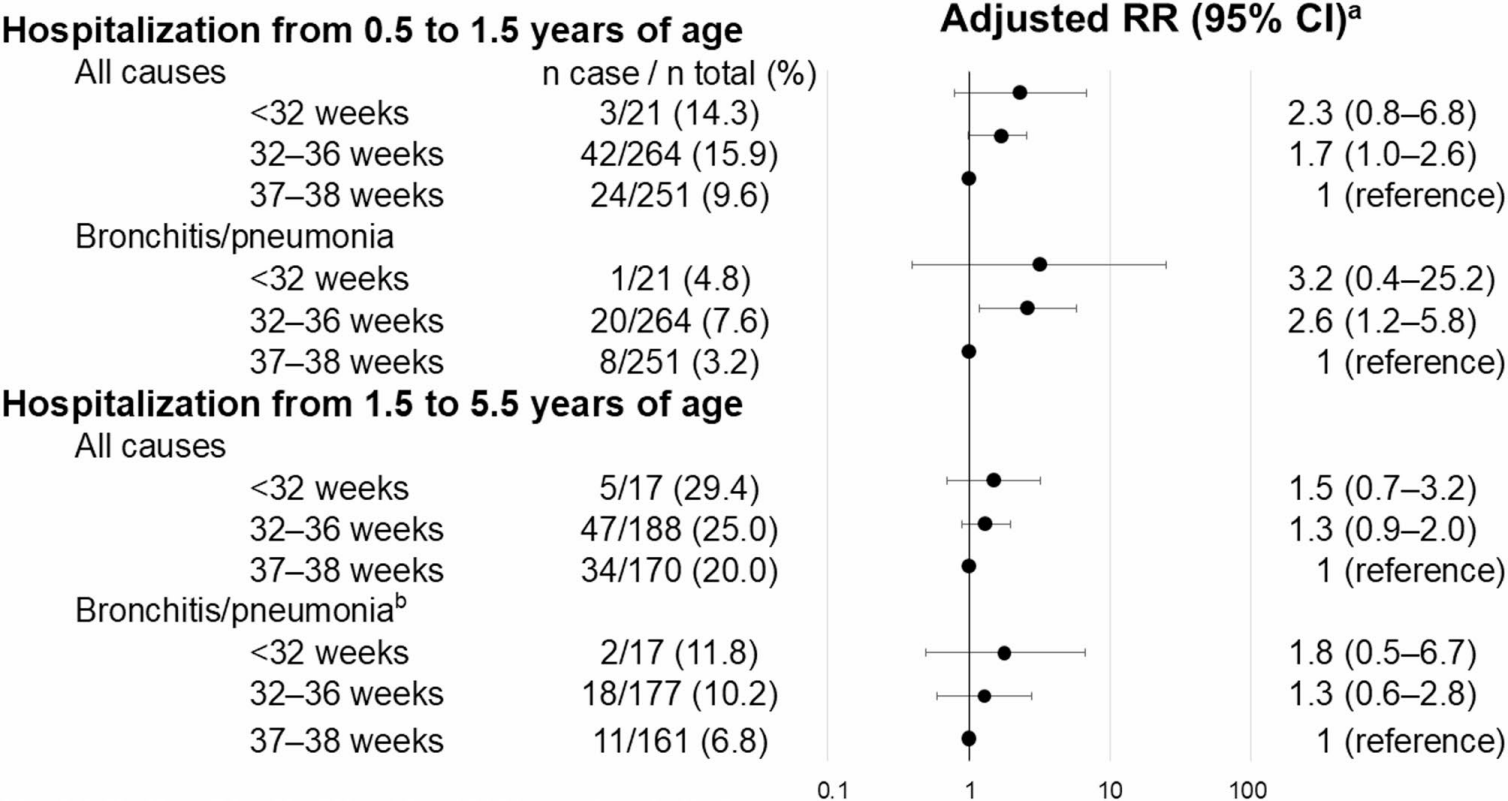
Adjusted RRs for associations between gestational age categories and hospitalization among twins. CI, confidence interval; RR, risk ratio.^a^Adjusted for sex, same-sex pair, small for gestational age, parity, maternal age at delivery, maternal smoking, maternal educational attainment, and place of birth and residence; ^b^we omitted place of birth and residence from the confounders because convergence was not achieved.

The associations between gestational age categories and hospitalization during infancy and early childhood are shown in Fig. 2. Children born moderately and late preterm had a higher risk of hospitalization during infancy than those born early term. Children born very preterm also showed a higher point estimate of the RR for hospitalization during infancy, although the 95% CI was wide, than the other groups of children. Specifically, the adjusted RRs for all-cause hospitalization between 0.5 and 1.5 years of age were 2.3 (95% CI: 0.8–6.8) for children born very preterm and 1.7 (95% CI: 1.0–2.6) for children born moderately and late preterm compared with those born early term.

**Fig. 3:**
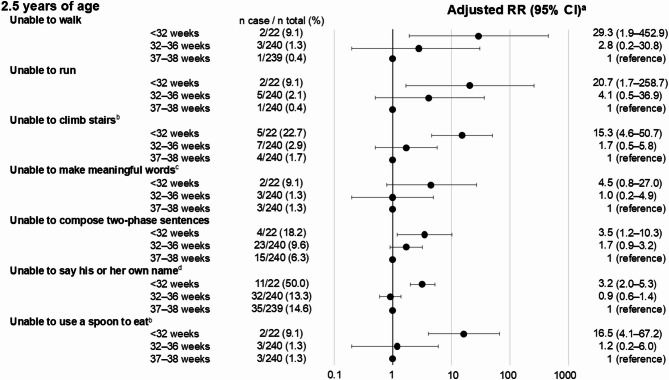
Adjusted RRs for associations between gestational age categories and behavioral development at 2.5 years of age among twins. Abbreviations: CI, confidence interval; RR, risk ratio. ^a^Adjusted for sex, same-sex pair, small for gestational age, parity, maternal age at delivery, maternal smoking, maternal educational attainment, and place of birth and residence; ^b^we omitted maternal age at delivery and place of birth and residence from the confounders because convergence was not achieved; ^c^we omitted place of birth and residence from the confounders because convergence was not achieved; ^d^we omitted same-sex pair, maternal age at delivery, and place of birth and residence from the confounders because convergence was not achieved.

Figure 3 shows the associations between gestational age categories and behavioral development at 2.5 years of age. Children born very preterm had a higher risk of unfavorable behavioral development than those born early term. Additionally, children born moderately and late preterm showed a higher risk in some questions, although the 95% CI was wide, than those born early term. In particular, the adjusted RR for being unable to compose two-phrase sentences was 3.5 (95% CI: 1.2–10.3) for children born very preterm and 1.7 (95% CI: 0.9–3.2) for children born moderately and late preterm compared with those born early term.

**Fig. 4:**
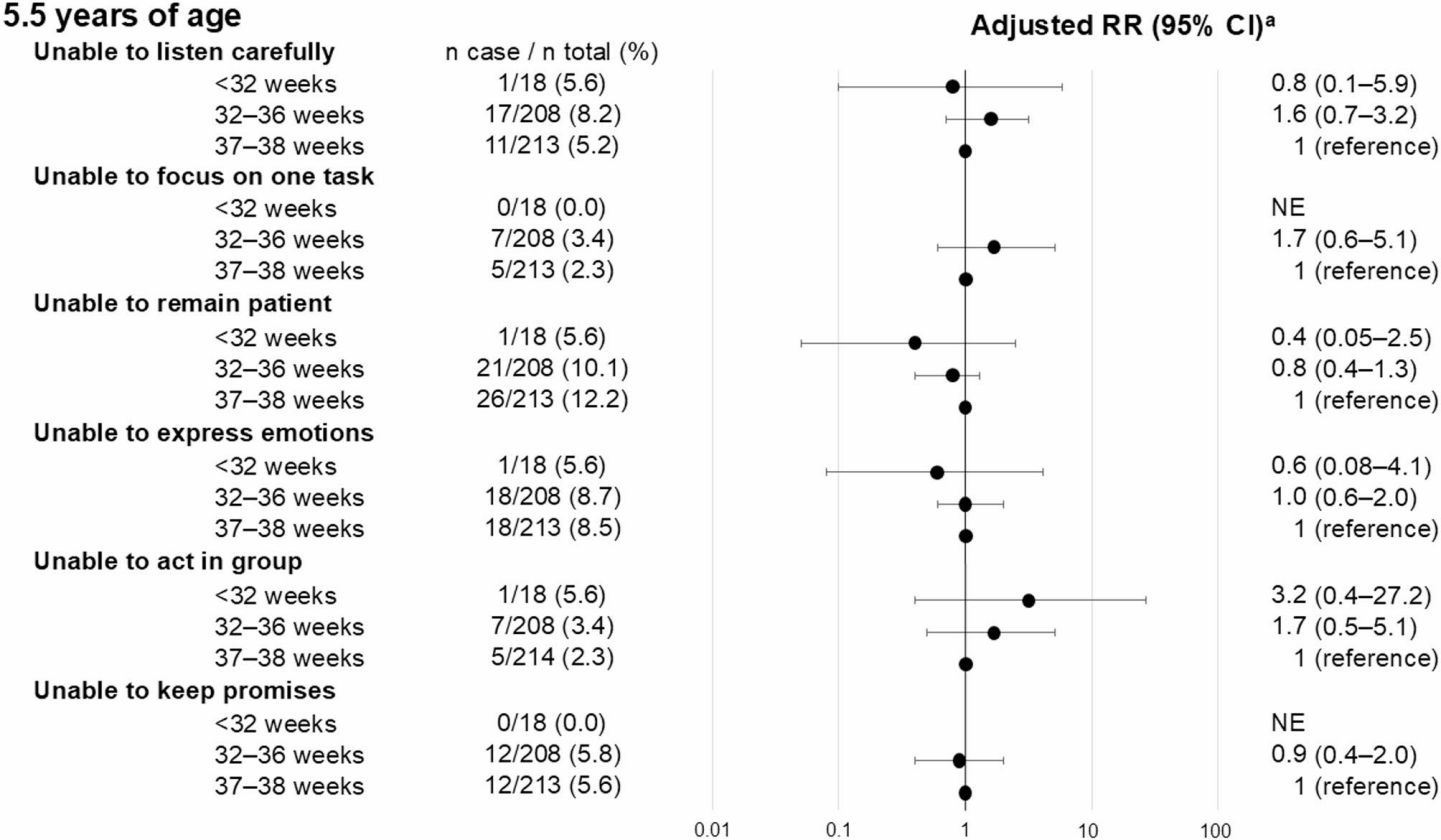
Adjusted RRs for associations between gestational age categories and behavioral development at 5.5 years of age among twins. CI, confidence interval; RR, risk ratio. ^a^Adjusted for sex, same-sex pair, small for gestational age, parity, maternal age at delivery, maternal smoking, maternal educational attainment, and place of birth and residence.

Figure 4 shows the associations between gestational age categories and behavioral development at 5.5 years of age. Some questions showed a slightly increased risk in children born very preterm and moderately to late preterm than those born early term, but no consistent trend was identified.

**Fig. 5:**
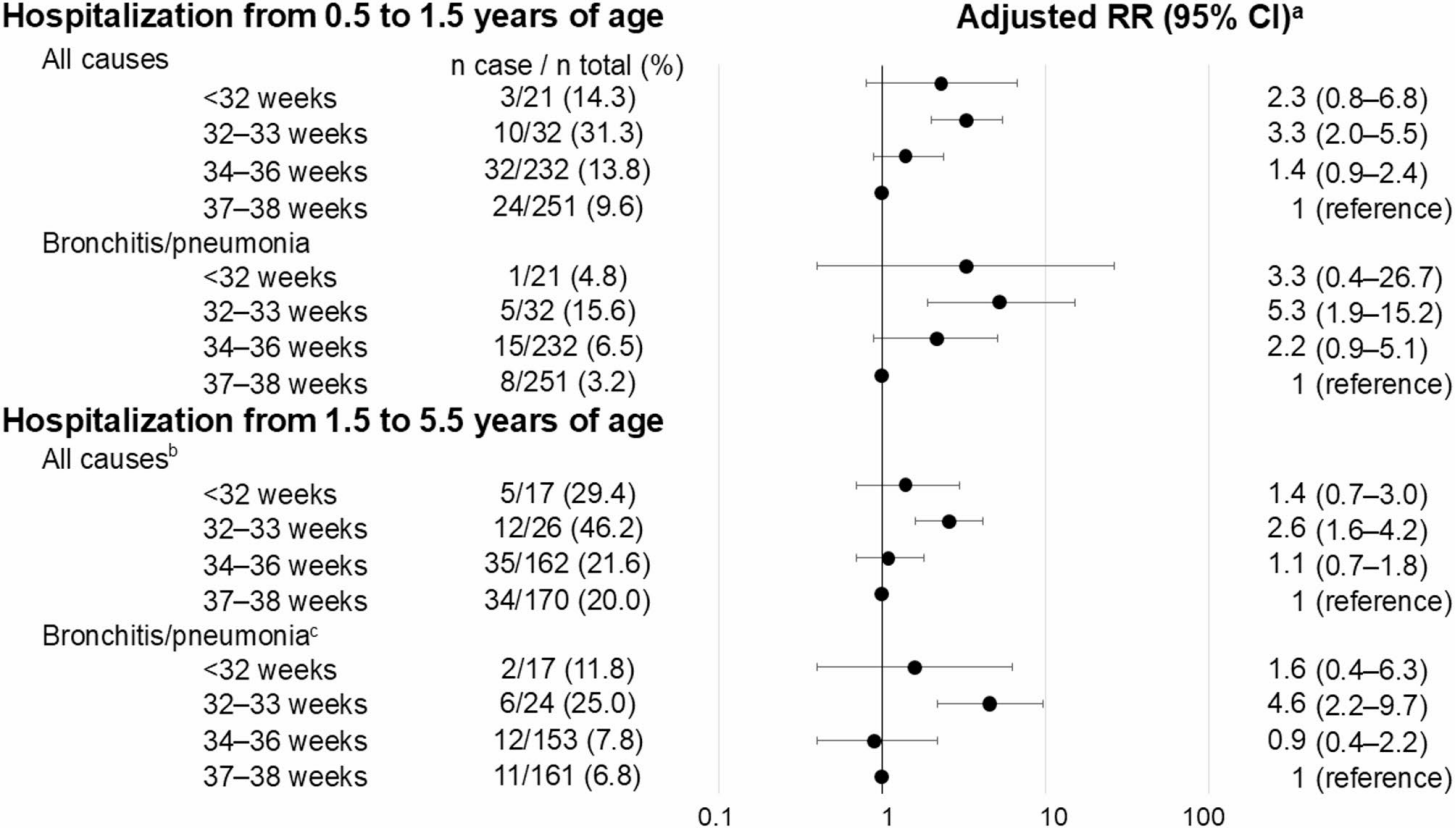
Adjusted RRs for associations between more detailed gestational week categories and hospitalization among twins. Abbreviations: CI, confidence interval; RR, risk ratio. ^a^Adjusted for sex, same-sex pair, small for gestational age, parity, maternal age at delivery, maternal smoking, maternal educational attainment, and place of birth and residence; ^b^we omitted maternal smoking during pregnancy from the confounders because convergence was not achieved; ^c^we omitted place of birth and residence from the confounders because convergence was not achieved.

In the sensitivity analyses, the RR for hospitalization was higher for children born at 32–33 weeks than for those born at 34–36 weeks (Fig. 5). Similarly, the RR for unfavorable behavioral development at 2.5 years was higher in children born at 32–33 weeks compared with those born at 34–36 weeks (Supplementary Fig. 1). At 5.5 years, some measures showed a similar tendency, but overall trends were inconsistent (Supplementary Fig. 2).

## Discussion

Using a nationwide, population-based, longitudinal survey in Japan, we investigated the associations between gestational age and child health and neurodevelopment during infancy and early childhood among twins. Among twins born before 39 weeks of gestation, those in the preterm groups (< 32 weeks and 32–36 weeks) had a higher risk of poor child health and adverse neurodevelopment at 2.5 years of age than those in the early term group (37–38 weeks). This risk increased as the gestational age decreased. However, these differences in behavioral development between the groups were not observed at 5.5 years of age.

Few studies have examined the association between gestational age and long-term outcomes among twins. In addition to the two studies described in the Introduction, a population-based cohort study in Korea (*n* = 17,189 mothers) examined this association^25^. This previous study showed that twins born before 37 weeks of gestation between 2007 and 2010 had a higher risk of neurological or neurodevelopmental delay at 8–11 years of age than those born at 37 weeks. However, data on the association between gestational age and childhood hospitalization among twins remains scarce. Due to Japan’s universal health insurance system, the quality of medical care among participants in this study is relatively homogeneous, which may have reduced variability in hospitalization thresholds and healthcare access. Therefore, the risk of hospitalization for twins born preterm is unlikely to have been underestimated in the present study.

Using the 2001 cohort of the Longitudinal Survey of Babies in the 21 st Century, we previously identified a U-shaped relationship between gestational age and the risk of childhood hospitalization, with the lowest risk observed at 37 weeks of gestation^9^. In the present study, we excluded twins born at ≥ 39 weeks (*n* = 16) because of the trend toward earlier delivery of twins in Japan, where births at this gestational age have become rare. We found that birth at 37–38 weeks was associated with the most favorable prognosis. These findings are consistent with previous cohort studies^9,10,25^, suggesting that the balance between fetal maturation and the risks associated with preterm birth plays a critical role.

Even if the obstetric management has changed, the preferred gestational age for delivery of twins may not have differed from that reported in previous studies. Overall, our results support current clinical guidelines in various countries recommending delivery of twins at 37–38 weeks of gestation.

The lack of differences in behavioral development between the groups at 5.5 years of age may reflect the strong influence of environmental factors on behavioral outcomes. Early childhood is a critical period for brain growth and development, during which children are highly sensitive to their environment and interactions with those around them^27^. Evidence suggests that family context and socio-demographic factors play significant roles in early child development^28^. In addition, postnatal interventions, such as breastfeeding status, may also influence developmental delays in early childhood^24,29^ and were considered intermediate factors in this study. The Longitudinal Survey of Babies in the 21 st Century (2001 cohort), including singletons, also assessed the developmental status in preterm infants at 2.5 and 5.5 years of age using the same questionnaire as in the present study^20^. This analysis was conducted in the entire cohort, not limited to twins. In that cohort, the odds ratios of developmental delays were higher at 2.5 years of age but decreased by 5.5 years of age, with the risk diminishing in certain categories, especially among late or moderately preterm infants. Environmental influences, developmental catch-up, variations in sample size, children’s ages, and the instruments used to assess behavioral issues may all have contributed to these discrepancies.

There are several limitations to this study. First, we lacked data on chorionicity and amnionicity classifications of twins, which influence neonatal health. Because the Longitudinal Survey of Babies in the 21 st Century does not include information on chorionicity, we could not account for its potential influence in this study. Although we adjusted for gender concordance as a proxy, this may not fully address the impact of chorionicity. In addition, due to data limitations, we were unable to examine potential interaction effects between preterm birth and sex differences among twins, which remains an important area for future research. Second, the questions used to evaluate behavioral outcomes were not externally validated, raising concerns about ambiguity. Nonetheless, these questions were used in previous studies in which expected associations were observed between unfavorable outcomes and gestational age, small-for-gestational-age status, and breastfeeding^20,21,24^. Third, although we adjusted for a wide range of potential confounders in the primary and sensitivity analyses, including socioeconomic status such as maternal smoking and educational attainment, residual confounding remains a possibility. An example of this possibility is that data on pregnancy complications and delivery modes, such as cesarean delivery, were only available for a few subjects. Moreover, other socioeconomic factors, as well as unmeasured parental or household factors—such as parental mental health, caregiving capacity, and access to early intervention services—may also influence the observed associations and were not accounted for in our analysis. Future research could benefit from incorporating this information for further adjustments. Fourth, the sample size in the present study is relatively small, particularly in the very preterm group, where wide confidence intervals raise concerns about statistical power. These limitations warrant caution when interpreting the estimates for this group. Fifth, the loss to follow-up is another limitation (Fig. 1). High-risk children whose mothers smoked, had lower education levels, and who were born preterm were more likely to be lost to follow-up (Supplementary Table 2), which may have resulted in an underestimation of the adverse effects of preterm birth on outcomes among twins. Finally, very preterm infants were more likely to have missing data on maternal educational attainment, which led to an underestimation of our findings as our analysis was restricted to complete cases.

## Conclusion

Our findings indicate that twins born preterm have a higher risk of hospitalization and adverse behavioral developmental outcomes during infancy than those born early term. These risks tend to increase as gestational age decreases. Our results support current clinical guidelines in various countries recommending delivery of twins at 37–38 weeks of gestation. Further research, including that on long-term outcomes, is essential to clarify how the timing of delivery in twin pregnancies influences outcomes, with particular attention to incorporating data on chorionicity and amnionicity.

## Supplementary Information

Below is the link to the electronic supplementary material.

Supplementary Material 1

Supplementary Material 2

Supplementary Material 3

Supplementary Material 4

## Data Availability

The datasets generated during and/or analyzed during the current study will be made available from the corresponding author upon reasonable request and with the approval of the Ministry of Health, Labour and Welfare of Japan.
