# Supplementary material for: Association between gestational age and child health and neurodevelopment in twins from a nationwide longitudinal survey in Japan
Source: Sci Rep. 2025 Nov 18;15:40608. doi: 10.1038/s41598-025-24186-2 (PMC12627776; doi:10.1038/s41598-025-24186-2)
Supplement: Supplementary file 1 — Supplementary Material 1 [file 41598_2025_24186_MOESM1_ESM.docx]

| Supplementary Table 1. Characteristics of infants with and without missing confounder data | | | |
| --- | --- | --- | --- |
|  | Infants without missing confounder data | Infants with missing confounder data | All |
|  | (N = 549) | (N = 115) | (N = 664) |
| Sex |  |  |  |
| Male | 270 (49.2) | 60 (52.2) | 330 (49.7) |
| Female | 279 (50.8) | 55 (47.8) | 334 (50.3) |
| Sex concordance of twin pairs |  |  |  |
| Same sex | 401 (73.0) | 83 (72.2) | 484 (72.9) |
| Different sex | 148 (27.0) | 32 (27.8) | 180 (27.1) |
| Parity |  |  |  |
| Primipara | 293 (53.4) | 55 (47.8) | 348 (52.4) |
| Multipara | 256 (46.6) | 60 (52.2) | 316 (47.6) |
| Small for gestational age | 165 (30.1) | 41 (35.7) | 206 (31.0) |
| Maternal age categories |  |  |  |
| <25 years | 26 (4.7) | 22 (19.1) | 48 (7.2) |
| 25–34 years | 320 (58.3) | 68 (59.1) | 388 (58.4) |
| ≥35 years | 203 (37.0) | 25 (21.7) | 228 (34.3) |
| Maternal smoking during pregnancy | 26 (4.7) | 24 (20.9) | 50 (7.5) |
| Maternal educational attainment |  |  |  |
| University graduate or higher | 146 (26.6) | 0 (0.0) | 146 (26.6) |
| Vocational school/junior college graduate | 261 (47.5) | 0 (0.0) | 261 (47.5) |
| High school graduate or below | 142 (25.9) | 0 (0.0) | 142 (25.9) |
| Residential area |  |  |  |
| Wards | 178 (32.4) | 24 (20.9) | 202 (30.4) |
| Cities | 317 (57.7) | 81 (70.4) | 398 (59.9) |
| Towns and villages | 54 (9.8) | 10 (8.7) | 64 (9.6) |
| Gestational age category |  |  |  |
| <32 weeks | 22 (4.0) | 8 (7.0) | 30 (4.5) |
| 32–36 weeks | 271 (49.4) | 55 (47.8) | 326 (49.1) |
| 37–38 weeks | 256 (46.6) | 52 (45.2) | 308 (46.4) |

Categorical variables were described by number (%).
