# Supplementary material for: Association between gestational age and child health and neurodevelopment in twins from a nationwide longitudinal survey in Japan
Source: Sci Rep. 2025 Nov 18;15:40608. doi: 10.1038/s41598-025-24186-2 (PMC12627776; doi:10.1038/s41598-025-24186-2)
Supplement: Supplementary file 2 — Supplementary Material 2 [file 41598_2025_24186_MOESM2_ESM.docx]

| Supplementary Table 2. Characteristics of infants with and without follow-up data at 2.5 years of age | | |  |
| --- | --- | --- | --- |
|  | Infants with follow-up data | Infants without follow-up data | ALL |
|  | (N = 502) | (N = 47) | (N = 549) |
| Sex |  |  |  |
| Male | 242 (48.2) | 28 (59.6) | 270 (49.2) |
| Female | 260 (51.8) | 19 (40.4) | 279 (50.8) |
| Sex concordance of twin pairs |  |  |  |
| Same sex | 372 (74.1) | 29 (61.7) | 401 (73.0) |
| Different sex | 130 (25.9) | 18 (38.3) | 148 (27.0) |
| Parity |  |  |  |
| Primipara | 269 (53.6) | 24 (51.1) | 293 (53.4) |
| Multipara | 233 (46.4) | 23 (48.9) | 256 (46.6) |
| Small for gestational age | 150 (29.9) | 15 (31.9) | 165 (30.1) |
| Maternal age categories |  |  |  |
| <25 years | 24 (4.8) | 2 (4.3) | 26 (4.7) |
| 25–34 years | 279 (55.6) | 41 (87.2) | 320 (58.3) |
| ≥35 years | 199 (39.6) | 4 (8.5) | 203 (37.0) |
| Maternal smoking during pregnancy | 20 (4.0) | 6 (12.8) | 26 (4.7) |
| Maternal educational attainment |  |  |  |
| University graduate or higher | 134 (26.7) | 12 (25.5) | 146 (26.6) |
| Vocational school/junior college graduate | 247 (49.2) | 14 (29.8) | 261 (47.5) |
| High school graduate or below | 121 (24.1) | 21 (44.7) | 142 (25.9) |
| Residential area |  |  |  |
| Wards | 162 (32.3) | 16 (34.0) | 178 (32.4) |
| Cities | 290 (57.8) | 27 (57.4) | 317 (57.7) |
| Towns and villages | 50 (10.0) | 4 (8.5) | 54 (9.8) |
| Gestational age category |  |  |  |
| <32 weeks | 22 (4.4) | 0 (0.0) | 22 (4.0) |
| 32–36 weeks | 240 (47.8) | 31 (66.0) | 271 (49.4) |
| 37–38 weeks | 240 (47.8) | 16 (34.0) | 256 (46.6) |
| Categorical variables were described by number (%). | |  |  |
