# Supplementary figures and images for: Association between gestational age and child health and neurodevelopment in twins from a nationwide longitudinal survey in Japan
Source: Sci Rep. 2025 Nov 18;15:40608. doi: 10.1038/s41598-025-24186-2 (PMC12627776; doi:10.1038/s41598-025-24186-2)

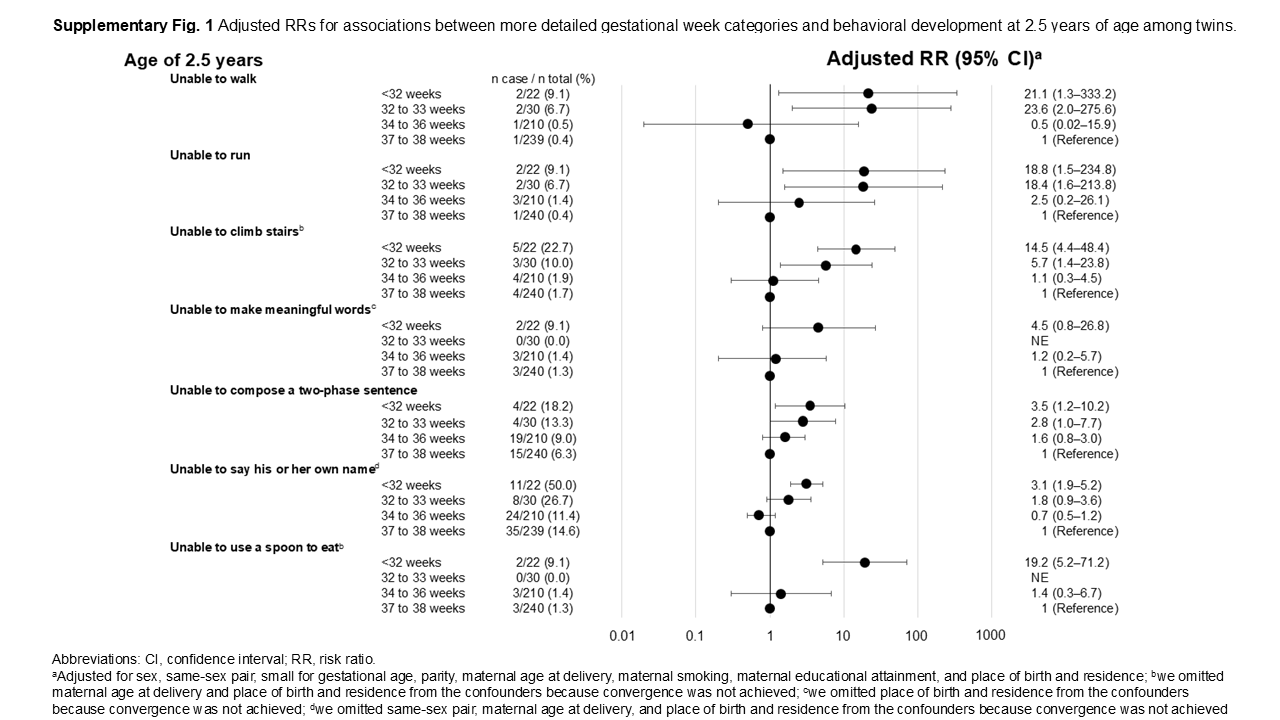

Supplement: Supplementary file 3 — Supplementary Material 3 [file 41598_2025_24186_MOESM3_ESM.tif]

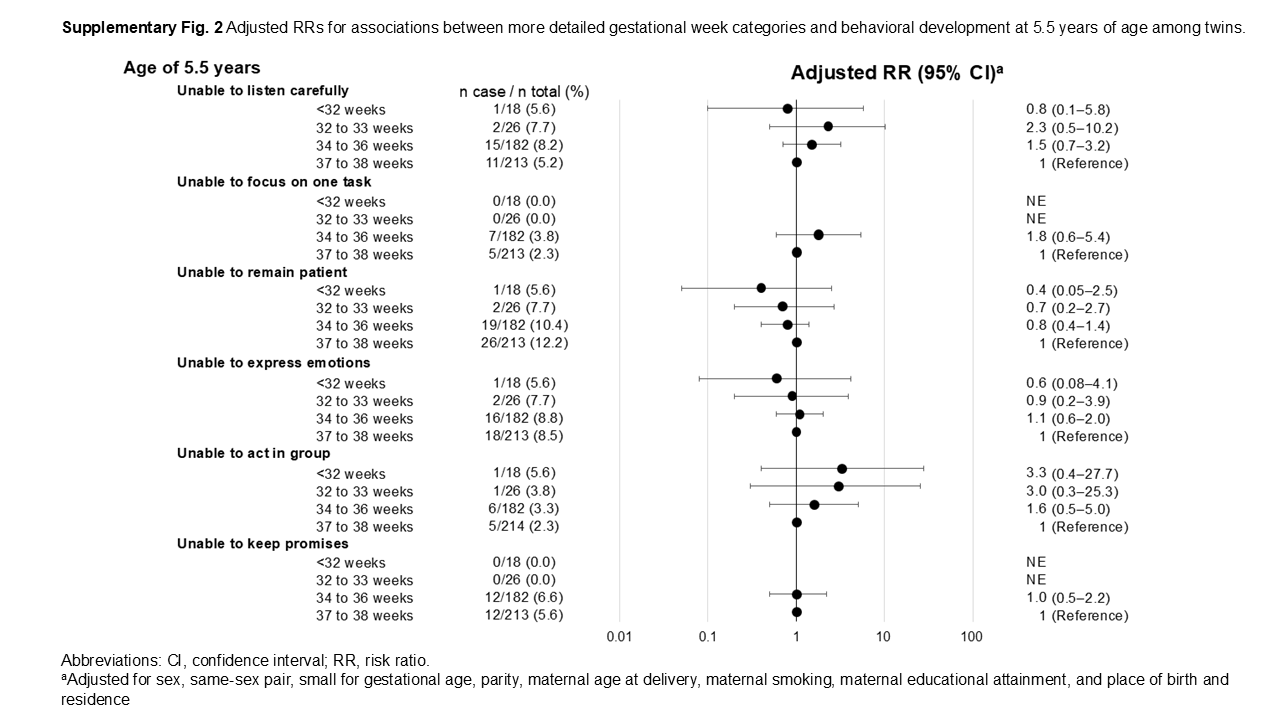

Supplement: Supplementary file 4 — Supplementary Material 4 [file 41598_2025_24186_MOESM4_ESM.tif]
